# Development of the Translating Allied Health Knowledge (TAHK) Framework

**DOI:** 10.15171/ijhpm.2019.23

**Published:** 2019-04-29

**Authors:** Danielle Hitch, Genevieve Pepin, Kate Lhuede, Sue Rowan, Susan Giles

**Affiliations:** ^1^ Occupational Therapy, Health & Social Development, Deakin University, Geelong, VIC, Australia.; ^2^ Occupational Therapy, North West Mental Health, Melbourne, VIC, Australia.; ^3^ Occupational Therapy, Barwon Health, Geelong, VIC, Australia.; ^4^ Occupational Therapy, Western Health, Melbourne, VIC, Australia.

**Keywords:** Allied Health Occupations, Allied Health Personnel, Knowledge Translation, Implementation Science, Knowledge Exchange

## Abstract

**Background:** While evidence-based practice is a familiar concept to allied health clinicians, knowledge translation (KT) is less well known and understood. The need for a framework that enables allied health clinicians to access and engage with KT was identified. The aim of this paper is to describe the development of the Translating Allied Health Knowledge (TAHK) Framework.

**Methods:** An iterative and collaborative process involving clinician and academic knowledge partners was utilised to develop the TAHK Framework. Multiple methods were utilised during this process, including a systematic literature review, steering committee consultation, mixed methods survey, benchmarking and measurement property analysis.

**Results:** The TAHK Framework has now been finalised, and is described in detail. The framework is structured around four domains – Doing Knowledge Translation, Social Capital for Knowledge Translation, Sustaining Knowledge Translation and Inclusive Knowledge Translation – under which 14 factors known to influence allied health KT are classified. The formulation of the framework to date has laid a rigorous foundation for further developments, including clinician support and outcome measurement.

**Conclusion:** The method of development adopted for the TAHK Framework has ensured it is both evidence and practice based, and further amendments and modifications are anticipated as new knowledge becomes available. The Framework will enable allied health clinicians to build on their existing capacities for KT, and approach this complex process in a rigorous and systematic manner. The TAHK Framework offers a unique focus on how knowledge is translated by allied health clinicians in multidisciplinary settings.

## Background

The concept of ‘allied health’ describes health disciplines other than doctors and nurses, and while definitions vary, the term has become increasingly prevalent in policy and practice in recent decades.^1,2^ In Australia, this umbrella term covers 22 professions including art therapy, chiropractic, dietetics, pharmacy, occupational therapy, physiotherapy, optometry, psychology, social work and speech pathology.^3^ Each of these professions brings with it a unique set of specialised knowledge, scope, philosophical basis and practice culture.

The concept of evidence-based practice has been part of healthcare for more than 20 years, with the original definition by Sackett and colleagues focused on decision-making regarding the care of individual patients.^4^ While initially limited to methods within the hierarchy of quantitative evidence, understandings of evidence-based practice gradually expanded to become more inclusive of non-medical areas of practice and broader forms of evidence.^5^ Rather than basing their evidence on knowledge which may not be fully fit for purposes, some allied health disciplines have advocated for an evidence informed approach which more explicitly incorporates clinical knowledge and patient preferences.^6^

However, evidence based or informed practice has not quite lived up to its promise in allied health. The barriers and challenges facing allied health clinicians when they attempt to translate knowledge into practice are well known. Allied health clinicians of all disciplines have repeatedly cited a lack of time, skills and access to peer and technological resources; uncertainty about the clinical meaning of research, and perceived lack of priority as non core business as key inhibitors.^7-10^ All allied health clinicians receive training in evidence-based practice prior to qualifying, and attitudes towards these practices are consistently reported to be positive, however these skills and beliefs do not automatically result in the translation of evidence into practice.^11,12^ The process of adapting and translating evidence generated elsewhere into the local context continues to be the missing link.

A recognition of these unresolved challenges has prompted a shift in the way allied health clinicians conceptualise the application of knowledge to practice. Knowledge translation (KT) is defined as a “dynamic and iterative process that includes the synthesis, dissemination, exchange and ethically sound application of knowledge to improve health, provide more effective health services and products, and strengthen the health care system.”^13^ This definition underscores the dynamic (rather than linear) and iterative (rather than procedural) approach required to successfully translate knowledge in a manner that meets the needs of diverse patients, clinicians and health organisations. The term ‘knowledge,’ rather than ‘evidence,’ also acknowledges the potential for utilising non-empirical information (such as policies, theoretical frameworks, patients and family lived experience). The purpose of KT moves beyond improving outcomes for individual patients, to initiatives that effect positive change in services and systems.

The discourse in allied health is currently in a state of transition, where references to evidence based or informed practice remain prevalent, but references to KT are increasing. While other terms (such as knowledge transfer, knowledge mobilisation, knowledge utilisation, and implementation science) do also appear sporadically, KT is currently the terminology of choice. However, there is significant diversity amongst the ways that KT is enacted within allied health, as reported in peer review literature over the past decade.

## Current Practices in Allied Health Knowledge Translation

Current practices in allied health KT are influenced by both facilitators and barriers, which are specifically relevant to this workforce. One of the challenges to gaining an understanding of how KT is enacted in allied health is their frequent identification as an undifferentiated population. For example, both nurses and allied health clinicians completing online training around infection control were more likely to transfer that knowledge if they perceived organisational support,^14^ but findings specific to each of these groups were not reported. While this reflects the multidisciplinary environments that allied health clinicians work within, it can obscure their specific needs as a workforce. There are also barriers specific to allied health around the identification of appropriate and relevant knowledge for translation. The diverse disciplines and vocabularies used in KT has been found to have a negative impact on the search filters of one of the key databases in this field – the Cumulative Index of Nursing and Allied Health Literature,^15^ leading to suboptimal findings from evidence searches.

A commonly reported strategy to support KT in allied health is knowledge brokerage roles, which have been implemented in several services. A qualitative study of nursing and allied health knowledge brokers and their mentors, highlighted that while these roles were valued they were not specifically recognised within organisations, which made arranging support particularly difficult.^16^ A case study of knowledge brokerage from occupational therapy^17^ found these roles were experienced as supportive by clinicians, who also saw them as complementing their existing skills and knowledge in this area. Other forms of personal mentorship (both face to face and remote) have also been utilised in allied health clinicians to knowledge and skills in specific areas of practice.^18^

Clinical practice guidelines have also been developed to enable allied health clinicians to enact best practice,^19-21^ as part of broader programs of KT strategies. Warner et al^22^ found that short narrative presentations of guidelines (omitting study characteristics such as design and sample size) did not provide sufficient detail to allied health clinicians to translate knowledge into practice. The effectiveness of clinical practice guidelines as a strategy to produce changes to patient outcomes remains uncertain,^23^ and allied health clinicians also draw upon a wider range of knowledge than those included in clinical practice guidelines.

Knowledge sharing initiatives like outcome measure databases,^24^ wikis and shared repositories,^25^ online decision-making support systems^26,27^ and evidence alert systems^28^ have also been implemented to support allied health clinicians. Keeping these resources up to date has been flagged as a potential barrier to use,^24^ as this task is time and resource intensive. However, the simple provision of information, even when synthesised in an accessible format, is insufficient to support effective KT.^20^ A systematic review of KT strategies used by 5 allied health professionals (dietetics, occupational therapy, pharmacy, physiotherapy, and speech pathology) identified an overreliance on educational strategies^29^ as a sole means for promoting KT. Multifactorial KT strategies are now beginning to emerge (such as the strategies used by Imms et al^30^), as understanding of the concept matures. Increasing reference is also being made to theoretical models and frameworks of KT, as allied health seeks to understand what does and does not work in practice.

## Theories and Frameworks to Guide Allied Health Knowledge Translation

A range of theories and frameworks are being utilised in allied health to guide KT. The Knowledge to Action process has been employed in several allied health KT initiatives.^28,31^ An advantage of process models such as this, are their ability to support a systematic approach to KT.^32,33^ However, they do not identify or explore the relationship between determinants that can impact on process, or provide insight into aspects of implementation. Examples of non-process frameworks applied to allied health include the Promoting Action on Research Implementation in Health Services (PARIHS) framework,^22^ and Pathmans’ awareness-to-adherence model.^12^ Classic theories which have arisen from other fields have also been utilised to support KT in allied health, including theories of change^34^ and Rogers diffusion of innovation theory.^13,27,35^

With the plethora of theories and frameworks available, selecting the most suitable one to use is a complex task. Birken et al^36^ found no meaningful consensus around the criteria which should be utilised when selecting an appropriate theory or framework, but recommended the transparent reporting of why a theory has been selected particularly in regards to empirical support, application to a specific setting/population and power/testability. However, as highlighted by Greenhalgh,^37^ no single theory, framework or model is likely to be sufficient to provide everything an allied health clinician needs to translate knowledge into their practice within their specific context. Nor can it be assumed that any of the currently available theories necessarily meet the specific needs of the allied health workforce.

## Rationale for the Translating Allied Health Knowledge Framework

The Translating Allied Health Knowledge (TAHK) Framework was developed in response to an identified need for allied health specific guidance for successful KT. An understanding of, and engagement with, practice contexts is fundamental to successful KT in health.^38^ To date, none of the KT frameworks in health have been developed within a specifically allied health context. Of the 35 frameworks reviewed by Nilsen,^33^ the majority originated from medicine or nursing, with others arising from non-health disciplines like education and management. Allied health clinicians work within a practice context that is discernably and significantly different than the larger professions of medicine and nursing, and which requires a tailored approach.

A key feature of the allied health practice context is its interdependence with other disciplines and professionals. Previous studies have demonstrated that doctors and nurses utilise allied health clinical input to meet their own disciplinary needs, but may not be fully aware of the unique disciplinary roles and skills of their allied health colleagues.^39^ While allied health clinicians have a degree of autonomy, they frequently work within multidisciplinary services, and are often single clinicians engaging with multiple interfaces across teams (and sometimes care sectors).^40^ While allied health clinicians have autonomy within their own practice, other team members may be gatekeepers for referrals. Given their embedded roles, all allied health KT is likely to have a broader impact on the practices of colleagues. A recent review found that the contribution of allied health to quality care can be maximised through connection and contextualisation from an integrated care perspective.^41^ Collaboration and interdisciplinary working is the default setting for allied health KT, and this necessarily increases the complexity of the task.

Allied health KT is also influenced by the characteristics of the knowledge available for translation. The majority of allied health interventions are complex, which presents design and evaluation challenges which often required both outcome and process evaluation to support translation.^42-44^ The gold standard randomised controlled trial is not often a suitable methodology in allied health, and several allied health professions explicitly challenge positivist approaches to knowledge.^20,34^ In some areas of the allied health practice context, knowledge has to be generated before KT can even begin, due to sparse or non-existent evidence.

KT in allied health can therefore entail a longer process, which includes the conduct of research as a first step. This is tacitly acknowledged in the frequent calls for clinicians to partner with researchers, or undertake research training, as a key KT strategy.^34,45^ Allied health managers have also highlighted difficulties in finding directly relevant research for their specific context, leading to the translation of knowledge derived from benchmarking against other services.^46^ With the emergence of new methodologies such as realist evaluation,^47^ and greater deployment of mixed methods, allied health KT can adopt more rigorous approaches which are fit for its practice context. There is also widespread acknowledgement that a stronger research culture needs to be incorporated into allied health.^48^ While medical and nursing research positions have been in place for many decades, equivalent allied health positions are a relative novelty. Allied health KT is therefore at a different stage, and potentially on a different pathway, of development.

A key tension within the allied health practice context is between the preservation of diversity, and the adoption of a unifying professional identity. There are some common elements that are broadly relevant for all allied health clinicians. The allied health workforce is strongly feminised (although gender proportions vary between disciplines), and there is a high proportion of part time positions.^49^ All allied health clinicians have core, secondary and other tasks which broadly fall into the categories of assessment, therapy, education and manufacturing.^50^ While the term ‘allied’ originally referred to being ‘allied to medicine,’ this has now shifted towards a stronger perception of being allied to each other and to the communities they serve.^51^

However, there are also multiple sub-cultures within allied health, particularly at a disciplinary level,^52^ which can be a source of both healthy competition and conflict. As noted by Scott et al,^29^ the nature and scope of work within these distinct professions means that KT strategies and initiatives may not necessarily be transferable within the overarching allied health practice context. Allied health clinicians have multiple identities as discipline members, allied health clinicians and team members to negotiate as they work to translate knowledge into practice.

Further evidence for the unique features of the allied health practice context is provided by the adaptation of existing KT theories to meet its needs. For example, Metzler and Metz^53^ proposed an adapted version of the Knowledge-To-Action Process model for occupational therapists which explicitly highlighted occupation as the core concept. Thingpen et al^54^ constructed a process developed by allied health practitioners to meet their needs for rapidly synthesised and accessible knowledge about violence prevention initiatives, drawing on 3 existing knowledge transfer and exchange models.^55-57^ Such adaptations are not in themselves unusual in KT, as all health professionals work within varied contexts.^12^ However, they do indicate that none of the existing theories and frameworks were a tailored fit for the allied health practice context.

The recognition of this gap led to the development of the TAHK Framework, which will now been described. The overall aim of developing the TAHK was to co-produce a framework describing determinants that influence KT outcomes in allied health.^33,58^ The objectives for this framework included a high degree of accessibility and useability, and relevance to all allied health disciplines.

## Approach to Development

Multiple methods were used in the development of the TAHK Framework, driven by an overarching commitment to co-create with allied health clinicians. This process alternated between phases of development and evaluation, over a 5-year period. As highlighted in the International School on Research Impact Assessment statement,^59^ mixed methods using a variety of data sources is considered the optimal approach to assessing impact in the real world, and a range of data sources were utilised in the development of the TAHK. The development of the TAHK will now be summarised, with further detail about each step in this process available as Supplementary file 1.

An initial draft of the TAHK framework emerged from a process of reflection undertaken by a steering committee, all of whom were from the discipline of occupational therapy. This process began with a literature review of evidence about allied health KT, from which the first author synthesised key themes and findings. KT was identified as a complex activity, with multiple dimensions and determinants. An overarching conceptual structure was sought to organise this complexity and allow for analysis of relationships within the phenomenon of KT. The Pan Occupational Paradigm (POP)^60^ seeks to explain the phenomenon of human activity across 4 dimensions - doing, being, becoming and belonging. As a paradigm, it presents broad assumptions and perspectives, as an articulation of values and philosophy.^2,3^ While the POP originated from occupational therapy and occupational science, its focus on human activity is relevant to all allied health disciplines. Finally, a draft aligning these key themes and findings with the domains of the POP was discussed by the committee in reference to their lived experience as allied health clinicians. Several additions and modifications were made as a result, leading to the initial draft of the TAHK.

This draft then became the basis of a consultation process with knowledge partners. The TAHK framework was introduced in a 45-minute professional development seminar, which was available in face to face and video formats. A total of 37 allied health clinicians completed the seminars and provided feedback on the draft TAHK framework. The majority were female clinicians (n = 33, 89.19), employed as occupational therapists (n = 33, 89.19%), and/or working in community settings (n = 21, 56.76%). The domains and factors of the draft Framework were generally considered important, and most participants (n = 25, 67.57%) indicated an intention to use it to inform their practice. The main strengths of the TAHK framework identified were its multidimensionality and inclusiveness, while more clarity in terminology and examples of application to practice were identified as areas for improvement.

The findings of this consultation were used to consolidate the draft TAHK framework, and the provision of resources for application became the focus of the next phase of development. To gather examples of how the TAHK might align with allied health practice, the developers decided to investigate the use of a benchmarking approach. A further round of consultation was then undertaken, using professional development seminars as a forum for completing a draft benchmarking tool based on the TAHK framework.

Each seminar ran for approximately 90 minutes, and they were attended by a total of 53 clinicians (including occupational therapists, physiotherapists, social workers, neuropsychologists and dieticians). Participants were asked to reflect on a KT activity they had undertaken in the past 12 months, or were currently undertaking, and work through the document. Three occupational therapy academics also provided feedback on the tool after receiving the content of the seminar in individual sessions with the first author. Nine clinicians and 3 academics (n = 12) subsequently provided feedback on the tool via a measurement property survey, with a further 8 also completing a qualitative interview. The findings from this feedback indicated most of the participants agreed the benchmarking tool was a relevant and valid measure of influential factors for allied health KT. Psychometric analysis also indicated the tool had reasonable levels of face validity, content validity (for all but 2 items) and acceptability, however useability needed improvement.

Three themes emerged from the qualitative data – “The Complexity of KT,” “Focusing on Process rather than Outcomes,” and “Tell Me More.” The TAHK Framework and benchmarking tool were reported to enable greater awareness of the complex nature of KT, and provided a structure for planning new activities or reflection on current activities. Clinicians were more focused on using the TAHK Framework to support process, than as a framework for measuring outcomes. Again, most participants expressed a desire for more information and support to enable them to use the TAHK Framework more effectively for KT. This feedback was again consolidated into the TAHK Framework, which was then finalised. Each aspect of the Framework.

## The Translating Allied Health Knowledge Framework

The purpose of the TAHK Framework as described here is to provide a description of the determinants and dynamics of allied health KT, and to guide clinicians in their efforts to plan and evaluate KT in the allied health practice context. It is anticipated the TAHK Framework will evolve over time, as the results of on-going research are used to refine the framework and develop supporting technology.

There are 4 core assumptions that underpin the TAHK Framework. Firstly, allied health KT is a complex activity, which clinicians need to do, want to do or are expected to do (individually or in a group), and which is a meaningful and purposeful aspect of their working lives. It requires conversing and translating between the various languages, mediums and cultures present across disciplines and settings. Allied health KT is relevant to the practice of all allied health clinicians, both pre and post qualification and across the lifespan of their career. Lastly, it is also assumed to involve the application of diverse forms of knowledge (both scientific and non-scientific) to everyday practice.

### Key Concepts

The TAHK Framework defines KT as the activities that apply relevant knowledge into everyday allied health practice, to improve outcomes for patients, carers, clinicians and the health care system as a whole. This definition was derived from that provided by Straus et al,^13^ but emphasises the diversity of knowledge available and its active application into practice. Along with its status as the preferred terminology in allied health, KT was chosen as the key term due to its emphasis on the act of communicating between languages, mediums and cultures. The ‘lost in translation’ aspect of applying knowledge to practice is often in the foreground when speaking to allied health clinicians, where they reflected on the impact of the practice context factors on the success or failure of their KT efforts. The reference to knowledge, rather than research, also acknowledges that allied health clinicians apply a range of evidence and information to practice including policies, audit outcomes, guidelines, theory and patient/carer feedback.

As a result of the development process undertaken for the TAHK Framework, the terminology used within it has also been modified to increase accessibility across all allied health disciplines and align more closely with the previously stated core assumptions. The original framework used the terms ‘knowledge broker’ and ‘knowledge user.’ While knowledge broker is a common term in KT literature,^61^ and has been utilised in previous allied health research,^17^ it was perceived as problematic during knowledge partner consultation. Clinicians frequently stated that they did not understand what these terms meant, and could not see their relevance to practice. When referring to the individuals who drove KT activities in practice, they more usually referred to senior clinicians and/or others in formal positions of organisational power. Therefore, the term ‘knowledge broker’ has been replaced with ‘leader’ in the TAHK Framework.

The development of the TAHK Framework was facilitated by a genuine partnership between the authors, clinicians and academics, where the contributions of each group has resulted in changes and amendments over time. While ‘knowledge users’ is the prevalent term in KT literature,^62^ it was not reflective of the power relationships experienced during the development of the TAHK Framework, which reflected a process of co-production. Therefore, the term has been amended to ‘knowledge partner,’ to acknowledge everyone’s ability to create and apply new knowledge.

### Framework Structure

‘Doing’ is now ‘Doing Knowledge Translation’ to highlight the focus on performance of this activity. Being has been changed to “Social Capital for Knowledge Translation” to emphasise the collaborative nature of KT. Social Capital in this context refers to ‘the sum of the actual and potential resources embedded within, available through, and derived from the network of relationships possessed by an individual or social unit. Social Capital thus comprises both the network and the assets that may be mobilized through that network.’^63^ This concept was chosen to replace ‘Being’ due to its focus on the capacity within social networks, and the relationships between their constituents, to enable KT.

‘Becoming’ is now ‘Sustaining Knowledge Translation’ as the longer-term continuation of KT is an explicit aim of the TAHK Framework. Finally, ‘belonging’ has been changed to ‘Inclusive Knowledge Translation’ to reflect the commitment expressed by many participants to embed KT into all aspect of allied health practice (including health organisations, academia, training and education, government and regulatory bodies and the general community) and be inclusive of all stakeholders (particularly patients, consumers and carers).

The factors classified under each domain have been expanded and/or re-defined, in response to new knowledge from both consultations and emerging research in this area. For example, the factors relating to knowledge brokers continued to garner diverse responses from knowledge partners, and were therefore redeveloped in line with this feedback and more recent literature around the personnel involved in allied health knowledge brokerage.^16,64^

The TAHK Framework is depicted in a format that displays its components clearly and systematically (see Figure). To support clarity, each domain and its associated factors are presented separately. However, a statement at the bottom of the diagram reminds knowledge partners that all the factors are interdependent and impact on each other continuously when allied health knowledge is translated into practice. The streamlined design of this diagram reflects efforts to address the sense of being overwhelmed by the complexity of KT reported by the knowledge partners during the consultation phase. However, there were also consistent calls for more comprehensive information about the domains and factors, which will now been described in detail.

**Figure:**
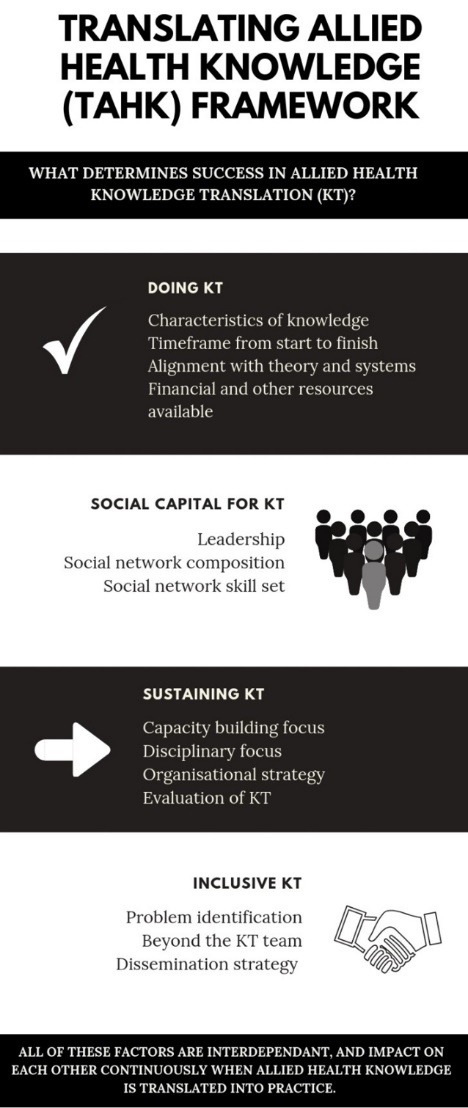
Diagram of the TAHK Framework. Abbreviations: KT, knowledge translation; TAHK, Translating Allied Health Knowledge.

## Doing Knowledge Translation

Doing allied health KT involves both active, explicit participation in KT tasks (ie, reviewing research, meeting with knowledge partners, etc), and tacit activities that contribute to the process (ie, reflecting on outcomes). The performance of allied health KT is influenced by 4 key factors, which impact upon how these activities are enacted.

### Characteristics of Knowledge

As stated previously, knowledge that is translated in the allied health practice context includes both scientific and non-scientific ways of knowing.^20,34^ During the second phase of knowledge partner consultation, allied health clinicians used the TAHK framework with projects that applied a range of knowledge, including research evidence, policy, consumer feedback, pedagogical approaches and feedback from an economic analysis. The type of knowledge being applied has a material impact on the approach to KT required. For example, the presence of clinical or best practice guidelines may support a systematic process of implementation undertaken largely within the health service.^65^ However, initiatives that include co-production or co-design with consumers involve completely different models and activities of KT.^66^ Allied health clinicians may choose to apply quantitative, qualitative, mixed method or non-scientific knowledge to change their practice, depending on service priorities. The availability of tools to support the critique the reporting of non-empirical knowledge, such as the checklists to appraise text and opinion pieces,^67^ and scoping reviews,^68^ support allied health clinicians to rigorously approach KT, regardless of the characteristics of the knowledge.

### Timeframe From Start to Finish

Time is often highlighted as an important factor (as both a facilitator and barrier to KT in allied health).^29,69^ Both the amount of time available to translate knowledge into practice, and the ability to reflect and revisit KT initiatives over time were identified as important by knowledge partnership during consultation. At the individual level, allied health clinicians reflected on how much time they have within their working week to engage in KT activities, which are often perceived to conflict with and be a lesser priority to clinical workloads.^70^ The timeframe of allied health KT projects determine their scope, particularly if an explicit deadline is present due to funding or staffing commitments. However, the iterative nature of KT means that KT projects may also be phases within a longer-term program of change and development. Allied health KT is therefore impacted upon by multiple timeframes throughout its process, all of which influence the realities of its performance.

### Alignment With Theory and Systems

The alignment of allied health KT with theories and frameworks, and with the systems to which the knowledge will be applied, also modifies its performance. Theoretical frameworks are frequently embedded within KT studies in allied health,^22,71,72^ and knowledge partner feedback indicated clinicians found these frameworks to be supportive of complex thinking and reflection. Not all of these theories and frameworks are related to KT, as some were discipline specific or addressed therapeutic approaches such as recovery and client centredness. The alignment of allied health KT with theories and frameworks may also support the transferability of findings from a specific service context, through reference to broader principles and concepts.

While theories and frameworks originate externally to the local allied health practice context, alignment to internal systems also influences how KT is performed. Integration with systems (such as electronic data management) has been found in previous research to facilitate allied health KT.^25^ Knowledge partners also identified that a lack of alignment between KT projects and systems such as supervision and training may be a barrier to their implementation. Embedding allied health KT in both external frameworks and internal systems increases the likelihood of its ongoing sustainability.

### Financial and Other Resources Available

A key and consistent theme to emerge from both the literature and knowledge partner consultation was the impact of resource availability on allied health KT. While there is some supporting evidence for the use of financial incentives to support allied health KT,^29,73^ knowledge partners report that the availability of sufficient funding for new or additional resources required is a crucial factor. Other resources and support for KT can include in-kind contributions, enabling interdisciplinary collaboration and the sharing of existing resources.^25,26,34^ A significant minority of knowledge partners reported lacking access to non-financial resources and support for KT, which were usually arranged and organised informally. Aside from resources specific to the knowledge being translated, allied health clinicians have also expressed a desire for resources that support their use of the TAHK Framework in practice.

## Social Capital for Knowledge Translation

The social capital invested in allied health KT, manifests in the roles people assume as part of the process, both individually and interpersonally, and the social network involved in project teams. There are 3 key factors related to the people involved in allied health KT, which impact on the resources available for success.

### Leadership

Leadership in allied health includes engagement with a range of contextual factors relevant to KT, including governance, professional standards and advocacy.^74^ The support and investment of senior and leadership clinicians is important to the success of KT in this practice context, and can play a significant role in supporting ongoing sustainability. However, allied health clinicians do not have to be in formal leadership positions to exercise leadership around KT. Champions for specific projects are frequently employed in allied health, and have been found to support the success of KT.^25,75^ The knowledge partners who were consulted during the TAHK Framework’s development had all led KT projects, but were drawn from all levels of clinical practice, from new graduates through to those in formal leadership positions. This factor in allied health KT is therefore a combination of local and project specific leadership (which is often informal), and formalised organisational leadership.

### Social Network Composition

The social network for an allied health KT project may be either single or multi-disciplinary.^20,26,28,34,76^ Mixed disciplinary groups are more prevalent in the literature,^28^ although the constituent disciplines are not always individually identified under the umbrella term of allied health.^15,27^ Knowledge partners also often mentioned patient, consumer and carer involvement in allied health KT project teams, although this is not common practice currently and is more often seen as an aspiration for the future. Social network analysis is a useful tool for understanding the complexity of KT in allied health,^12^ and can be employed to map the social capital available for a project both within a project team and beyond.

### Social Network Skill Set

Allied health clinicians at all levels of the workforce are engaged in KT,^25,26^ and knowledge partners consulted during the development of the TAHK framework were also diverse in their background and level of experience. While experience in the allied health practice context is invaluable to KT, older clinicians now in senior roles may have qualified before training in evidence-based practice and/or KT formed part of pre-registration education.^9^ Allied health KT is never an individual activity, and therefore the skills and experience available for a project is a product of the social network gathered to work on it. Reflecting on individual skill sets, and collective skill mix within a project team, clarifies areas where external assistance may be required and supports decision-making around how best to utilise the teams abilities.

## Sustaining Knowledge Translation

KT results in changes to personal and team practice,^34^ and the challenges to sustaining these changes over time (particularly when original project team members are no longer in the workplace) are well recognised. This domain of the TAHK Framework refers to the change over time that KT engenders, and how this relates to the goals, aspirations and motivations of all stakeholders. There are 4 factors that have been identified as important influences to the sustainability of allied health KT.

### Capacity Building Focus

Several initiatives to build the capacity of the allied health workforce to engage with KT are reported in the literature,^45^ and these strategies can have a positive impact on increasing participation.^75^ Knowledge partners consistent expressed a desire to learn more about KT, and capacity building was identified as the most important factor within the TAHK Framework during consultation. Allied health clinicians want to take an active part in KT, but do not currently feel confident or capable in the skills required for its success.^77^ Allied health KT must be based upon partnerships with the clinicians, which are inclusive of their active involvement in all aspects of the process.

### Disciplinary Focus

Both multidisciplinary and discipline specific approaches to allied health KT have been identified as effective,^25,26,28^ with much depending on the context and nature of the clinical issues being addressed. While many of the knowledge partners consulted focused their projects on their own discipline, most had the potential for multidisciplinary application (eg, falls prevention, sensory modulation). The use of different disciplinary approaches enables opportunities for both the consolidation of unique disciplinary identity and roles, and collaboration with colleagues more broadly. When undertaking multidisciplinary allied health KT, consideration must be given to communication between disciplinary languages and cultures to maximise the potential benefits of this approach.

### Organisational Strategy

The relationship of allied health KT to organisational strategy is related to its alignment with systems, and is a factor that specifically impacts upon sustainability. A key strategy for sustainable allied health KT discussed in the literature was discussing KT projects regularly in team meetings,^27,34,35^ to maintain its visibility. Many projects discussed by knowledge partners were explicitly aligned to organisational goals and/or a mission statement, which was noted to also support ongoing development such as grant applications and business case development. Allied health KT that is explicitly aligned to the organisations broader strategic direction is more likely to attract ongoing investment (of finances and other resources). Given allied health’s specific focus on functional analysis and health, and its established role in chronic disease management in the community,^41^ and the increasing priority given to these issues in global health, it is well placed.

### Evaluation of Knowledge Translation

Evaluating the outcomes of KT into practice is a key phase of the process,^78^ however it is rarely completed in the allied health practice context. Knowledge partners reported that the effort involved in implementing KT often left few resources or little time for evaluation of that implementation. The approach and methods to be employed in evaluation should be developed as part of the initial project planning, as without evaluation there is no evidence on which to base future development and funding requests. The TAHK Framework can support evaluation of allied health KT broadly, through its identification of key determinants for success that can be measured or targeted. An outcome measure associated with this framework is also currently under development, and will provide allied health clinicians with a further tool for evaluating their KT projects from both a process and outcome perspective.

## Inclusive Knowledge Translation

Finally, inclusive KT is the bridge between its inherent characteristics and the environment in which it operates. KT activities belong to a particular context, which includes factors such as service setting, professional cultures, local communities and temporal context. These belonging relationships are founded on reciprocity, mutuality and sharing, and the dimensions of doing KT, social capital for KT and sustainable KT all belong to a specific local context in which knowledge is applied. There are 3 factors that have been found to be influential for the inclusivity of allied health KT.

### Problem Identification

The problem to be addressed by KT should be collaboratively identified by knowledge partners,^79^ which should ideally include those who will be translating the knowledge and those who are impacted upon by that translation. This approach to problem identification was identified by knowledge partners as an important factor within the TAHK Framework, and is advocated more broadly by those who promote co-production and citizen science approaches.^80^ Meaningful involvement of stakeholders at this earliest phase of allied health KT projects also promotes greater engagement throughout the process, which was a consistent aspiration of knowledge partners.

### Beyond the Knowledge Translation Team

Due to the interdependent nature of allied health KT, its impact and influence extends far beyond the KT project team. Consultation and inclusion of knowledge partners and stakeholders beyond the project team provides a more comprehensive understanding of the context,^81,82^ and also requires flexibility around how their inclusion is facilitated. Within the health system, consulting beyond the KT team includes the possibility of liaising with non-clinical stakeholders, such as administration staff, volunteers and service staff. Including people beyond the project team may also involve collaboration with stakeholders beyond health, such as community groups, education providers, government agencies and research institutions.^81^ Knowledge partners reported that the inclusion of all stakeholders (including other staff, carers and patients) was very valued, and they were motivated to pursue this wherever possible.

### Dissemination Strategy

New knowledge and understandings derived from allied health KT projects must be shared to enable the border sector to benefit. Dissemination of KT projects in allied health enables collective learning, and in itself contributes to the knowledge base.^76,83^ The majority of definitions of KT provided by knowledge partners included direct reference to dissemination strategies such as publication and conference presentations. Similarly to evaluation, planning for disseminating allied health KT should commence in the earliest stages of a project, and include both formal and informal forums of communication.

## Critical Reflection on the Development of the TAHK Framework

The significant time invested in consultation with knowledge partners has enabled a comprehensive and inclusive approach to the development of the TAHK Framework, which greatly increases its chances of being applied to practice by allied health clinicians into the future. The final version of the framework is significantly different to the initial draft, and the allied health workforces at each of the health services that participated have played a significant role in its final composition.

From this process, the authors have learned that KT in allied health requires an investment of time, and that a longer-term approach can yield a more comprehensive understanding of the phenomenon. This was particularly useful in engaging with the core challenge of having a rigorous framework, which also has enough flexibility to encompass the diversity of allied health KT. For example; feedback from knowledge partners often included comments to ‘make it simpler,’ but this cannot be achieved without potentially losing the framework’s sensitivity to complexity. Through iterative consultation, the TAHK Framework is now able to strike a balance between detail and useability.

However, there were several challenges associated with this iterative process that also contributed to the extended timeframe of the framework’s development. While many allied health clinicians were keen to test and trial the framework, few consented to provide feedback via the associated research reported here. Several indicated they did not feel sufficiently qualified to offer an opinion (despite the resources and tools being designed for clinicians), and generally the formal evaluation of the TAHK was not seen as such a priority as experientially using it. Recruitment of allied health clinicians was also difficult in relation to potential allied health academic participants. An invitation to participate in the consultation process was distributed to every allied health registration course in the locality of the authors. While no participants consented to take part, several indicated interest in the framework and sent responses encouraging its on-going development. The well documented challenges associated with allied health research engagement,^84^ and emerging state of research culture in this area, continues to be a characteristic of this practice context and potential barrier to ongoing development.

However, the positive support experienced by the authors throughout the framework development process, despite the absence of participation in the associated research, indicates that allied health clinicians perceive a need for greater support and guidance to perform KT. A more creative approach to research design, which integrates data collection with participation in activities based on the TAHK Framework (rather than approaching these domains separately), could be a more successful approach to promote on-going clinician involvement in future development.

## The TAHK Framework in Relation to Other Theories and Frameworks Currently Available

The TAHK Framework articulates an understanding of the determinants of KT, which is specific to the allied health practice context. Most of the factors identified are not novel to this framework, but their identification as being particularly important to allied health is what makes the TAHK framework tailored to this specific workforce. Some of the domains of the TAHK also align with those identified in other KT frameworks. For example, the social capital domain of the TAHK shares some features with the Characteristics of Individuals domain of the Consolidated Framework for Implementation Research,^85^ and the inclusion of multiple forms of evidence is also shared by the PARIHS framework.^86^ Generally speaking, it is not the content of the TAHK Framework that makes it unique, but its structure.

However, the conceptualisation of inclusive knowledge as a domain (rather than a factor) is a discernably different feature of the TAHK Framework in relation to other theories and frameworks. Greater inclusion of consumers and carers, via methods based on the principles of co-production and co-design, have gained growing traction in KT recently, and is a key concept for allied health clinicians for both KT and their daily practice. No other currently available theories and frameworks within health foreground inclusive KT practice in a similar manner to the TAHK. Its core position in the framework, and validation during successive rounds of knowledge partner consultation, expresses both its allied health context and current trends in the KT field.

The TAHK Framework is presented here as one of many possible theories and frameworks available to support allied health KT. As a determinant framework, it can be used in conjunction with process models such as the Knowledge to Action process,^28,31^ to identify the factors which impact upon the steps taken and activities required to get knowledge into practice. It is also complementary to other determinant frameworks, such as the PARIHS and Consolidated Framework for Implementation Research, because it articulates a distinctly allied health vision of KT that could inform KT projects undertaken from the perspectives of those models.

The key contribution of the TAHK Framework is its ability to articulate how allied health clinicians understand KT, and what potential barriers and enablers are important to their experience of these activities. An allied health specific understanding of KT has been absent to date, and its formulation is influenced by this sectors particular culture, working conditions and values. As allied health clinicians become increasingly active in both research and KT, the TAHK Framework supports clearer communication of complex concepts between the various languages, mediums and cultures of allied health, and the broader health community.

## Limitations

There are several limitations to the TAHK Framework that the authors would also like to acknowledge. The major limitation to date has been the relatively low proportion of participants who were from disciplines other than occupational therapy. The lack of diversity within participants during the development of the framework could be interpreted as limiting the frameworks applicability to only the profession of occupational therapy. However, a clear and consistent theme throughout this process of development has been the multidisciplinary nature of allied health KT. Limiting the TAHK Framework to a single discipline would greatly reduce its applicability and usefulness with the allied health practice context. Subsequent development currently underway is including more diverse participants, and the TAHK framework is being utilised by a range of allied health clinicians outside of occupational therapy. The framework is intended to be continually updated and amended as new information becomes available, and there is therefore the potential for it to be amended should the current structure not meet the needs of all allied heath disciplines as intended.

The 3 health services involved in the development of were all located in the same region of an Australian state, which may have potentially introduced some geographical factors that may not be transferable to other locations. The sample size of allied health clinicians who responded to the consultation process was also relatively small, which may also have introduced bias.

## Conclusion

The comprehensive and iterative process of development described during the development of the TAHK Framework highlights the resources required to develop such frameworks from the ‘bottom up,’ but also supports its adoption into practice. In particular, the central role played by clinicians in the development of the TAHK Framework is distinctive and has been extremely valuable.

As asserted by Birken et al,^36^ empirical support is the most important criteria for the selection of implementation frameworks, closely followed by analytic level and its ability to be applied to a specific settings/population. Two of these 3 criteria have been met, as the TAHK Framework operates at the organisational level and has been developed specifically for allied health clinicians. The ongoing testing of how the TAHK Framework is applied to practice will provide the empirical support required to make the TAHK Framework a resource of choice for allied health clinicians.

KT enables allied health clinicians to provide quality and ethically sound health care to their patients, and thereby meet their obligations as health professionals within the modern health care system. It also offers an opportunity for them to mobilise their existing skills and abilities in effectively engaging with complexity, at both the individual patient and broader organisational levels. The TAHK Framework is a new resource, but it does not require a completely new skill set from the workforce it was designed for. The TAHK Framework will support allied health clinicians to build on their existing capacity to enact KT, using a systematic and evidence-based framework they themselves have helped to construct.

## Ethical issues

The study was approved by Melbourne Health Human Research Ethics Committee (QA2015017), Western Health Human Research Ethics Committee (QA2015017), and Barwon Health Human Research Ethics Committee (14/163).

## Competing interests

Authors declare that they have no competing interests.

## Authors’ contributions

DH, GP, KL, SR, and SG made a significant contribution to the conception or design of this study. DH and GP made a significant contribution to the acquisition, analysis and/or interpretation of the data. All authors made a significant contribution to the drafting this study and subsequent revisions. All authors have provided final approval for this version to be published and agree to be accountable for all aspects of the accuracy and/or integrity of this study.

## Authors’ affiliations

^1^Occupational Therapy, Health & Social Development, Deakin University, Geelong, VIC, Australia. ^2^Occupational Therapy, North West Mental Health, Melbourne, VIC, Australia. ^3^Occupational Therapy, Barwon Health, Geelong, VIC, Australia. ^4^Occupational Therapy, Western Health, Melbourne, VIC, Australia.

## Supplementary files


Supplementary file 1 contains the development of the TAHK with further details.

## Key messages

**Implications for policy makers**

- Development of knowledge translation (KT) frameworks and processes in partnership with the people who will both use and be impacted by their use increases their likelihood of adoption into practice.
- Allied health KT needs to strike a balance between a rigorous approach, flexibility and useability.
- The Translating Allied Health Knowledge (TAHK) Framework offers an evidence and practice based theoretical structure for the development of technologies and resources to support allied health clinicians.
- Allied health clinicians already possess skills and abilities in effectively engaging with complexity (at both individual patient and broader organisational levels), suggesting that a focus on capacity building, rather than skills acquisition, is more likely to be effective.

**Implications for public**

Allied health clinicians provide a wide range of services, all of which aim to restore and maintain optimal health and wellbeing in their patients. Each discipline approaches this task with unique specialised knowledge, scope, philosophical basis and practice culture. The knowledge that allied health clinicians draw upon to provide their services is therefore diverse and rapidly developing. This paper describes the development of a framework for allied health clinicians, which highlights the factors that can support them to effectively translate new knowledge into their practice. It has been developed over the past five years via the integration of several different methods and approaches, in conjunction with practicing allied health clinicians and academics. The Translating Allied Health Knowledge (TAHK) Framework may support allied health clinician to apply the latest knowledge from research, guidelines and policy to treatment, and ensure consumers are receiving the best possible quality of care.
