# Supplementary material for: Development of the Translating Allied Health Knowledge (TAHK) Framework
Source: Int J Health Policy Manag. 2019 Apr 29;8(7):412–23. doi: 10.15171/ijhpm.2019.23 (PMC6706979; doi:10.15171/ijhpm.2019.23)
Supplement: Supplementary file 1 — contains the development of the TAHK with further details. [file ijhpm-8-412-s001.pdf]

Supplementary file 1

Initial Draft Framework Development

A steering committee of experienced clinical leaders from three health services (both metropolitan and regional) was formed in 2014, to ground the development of the TAHK Framework in the allied health practice context and co-ordinate formal consultation with clinicians. The group met on three occasions, and also communicated by email throughout that year, as development progressed. Potential committee members from a range of allied health disciplines were invited, but the committee was eventually formed entirely of occupational therapists.

The first version of the TAHK Framework developed from both a process of reflection undertaken by this committee, drawing upon their own lived experience as allied health clinicians and a literature review of studies of allied health knowledge translation (KT) available at that time. Thirteen factors recognised as influential to allied health KT were identified through this process, and classified under one of four domains – doing, being, becoming and belonging.

The dimensions of doing, being, becoming and belonging[1] were derived from the Pan Occupational Paradigm, which seeks to explain the human experience of activity engagement.[1] Domain definitions were adapted from this theoretical framework,[2] with the dimensions having an interdependent and simultaneous influence on KT. These dimensions were conceptualised as a complex adaptive system, which may be influenced by both positive and negative feedback loops and where outcomes may not always be predictable.[3] KT was conceptualised as the interaction between performed activities (doing); personal and professional identity (being); change and development over time (becoming); and social and environmental contexts (belonging).

Initial Consultation with Knowledge Partners

Given existing evidence that workshops may effectively develop knowledge for allied health clinicians,[4] a professional development seminar introducing the draft TAHK Framework was formulated. This 45-minute session was available in face-to-face and video formats, and included blended delivery that combined didactic information with interactive activities. The topics presented included; introduction to KT; reflecting on the knowledge available for practice; KT strategies in allied health; overview of the TAHK Framework domains and a reflection on their potential relationship to practice. Three face-to-face professional development seminars were provided at two health services (n = 32), with other participants completing the session via video either individually or in small groups (n = 14).

Ethical approval was received from three health services for clinicians to complete a mixed methods questionnaire following the seminar (Service 1 & 2 QA2015017, Service 3 14/163), about their perceptions of KT and the TAHK Framework. The questionnaire comprised 18 questions, including demographic details, understanding of and

participation in KT activities, perceived relevance of the TAHK Framework, perceived barriers to KT, perceived positive and challenging aspects of the TAHK Framework, and likelihood of future use. All data collected from this convenience sample was analysed descriptively.

A total of 37 clinicians completed the questionnaire (total seminar participants = 46, response rate = 80.43%). The majority were occupational therapists (n = 33, 89.19%), although there were also participants from dietetics, physiotherapy, speech pathology and social work (n = 4). The majority worked in community settings (n = 21, 56.76%), and had practiced for an average of 10.51 years (SD 8.84). All but four were female. The vast majority (n = 35, 94.59%) offered a definition of KT, with content analysis highlighting its location at the local service level, the need for both dissemination and integration into practice, and that KT involves diverse practices.

Most clinicians reported undertaking KT on either a weekly (n = 17, 45.95%) or monthly basis (n = 13, 35.13%), and around one third (n=12, 32.43%) had worked with an identified knowledge broker or person with a specific remit to undertaken KT. All but two clinicians stated KT was very or extremely important to their practice. As shown in Table 1, the domains and factors within the draft TAHK Framework were generally considered important, although the background and training of knowledge brokers were not as great a priority or perceived obstacle. The majority of TAHK Framework factors were identified as minor to moderate barriers, although integrating KT within existing systems (such as supervision, training etc.) was a more significant barrier.

Table 1. Clinician Ratings of Fraft TAHK Framework Factors in Regards to Importance and Magnitude of Barrier

		Importance (0 = not important, 4 = extremely important)		Magnitude of Barrier (0 = none at all, 4 = major barrier)	
**Domain**	**Factor**	**Mean**	**SD**	**Mean**	**SD**
Doing	Timeframe of project	2.43	1.04	1.69	0.71
	Sources of support	2.86	0.98	1.83	0.94
	Integration with existing systems	2.86	1.00	2.03	1.13
	Perception of evidence	2.51	0.93	1.64	0.90
Being	Knowledge broker role	2.70	1.02	1.29	1.13
	Knowledge broker Background	2.11	1.13	1.11	1.08
	Knowledge broker Training	2.11	1.10	1.20	1.02
Becoming	Capacity Building of workforce	2.97	0.96	1.86	0.97

	Organisation Strategic Plans or directions	2.84	0.96	1.86	1.06
	Discipline Specific	2.38	1.16	1.29	1.05
Belonging	Workforce Involvement	2.73	0.90	1.83	1.04
	Patient / Consumer Involvement	2.81	0.88	1.37	0.94
	Identification of priorities	2.81	0.88	1.77	1.00

Around a third of clinicians (n=12, 32.43%) indicated the TAHK Framework would be very or extremely useful to their practice, while a similar proportion (n=14, 37.84%) rated the framework as useful. The main strength of the TAHK Framework identified was its multidimensionality, which clinicians indicated encouraged them to *"Think about different aspects of KT" (Occupational Therapist)* which *"provided more structure" (Speech Pathologist)* to their reasoning. The inclusiveness of the TAHK Framework was also valued, as clinicians indicated it could be *"transferable across settings" (Occupational Therapist),* and they respected the *"inclusion of all stakeholders and recognition of cultural adaptation into the health service" (Social Worker*). The occupational therapists surveyed recognised the disciplinary origins of the model (which were not explicitly stated), and indicated they found this beneficial; *"The incorporation of the framework into an already familiar model - believe this will allow clinicians to relate and draw on it more easily" (Occupational Therapist).*

Four challenging aspects of the TAHK Framework were also identified, beginning with the amount of information presented in the session. While the multidimensionality of the TAHK Framework was appreciated, it was also perceived by some as overwhelming; *"too many tables, info overload!" (Social Worker).* Some terms also needed further explanation, as they were unfamiliar; "*The formalised concept of the knowledge broker role is new to me*" *(Dietician).* Clinicians were keen to receive specific information about how to apply the TAHK Framework, as they wondered '*where to from here*?" *(Occupational Therapist).* Finally, a significant minority (n=9) reported they don't have sufficient resources to enact KT in their setting; "*resources and knowledge brokers don't exist*" *(Occupational Therapist).*

Clinicians were asked to provide additional suggestions or recommendations for improvements to the TAHK Framework, but few were offered. Several made comments specific to the delivery of the session. However, recommendations offered included setting up a buddying or mentoring system to support use of the TAHK Framework and providing specific case studies of its use in practice; "*specific examples need to be given as its quite confusing and unclear*" *(Physiotherapist).* Many clinicians (n=25, 67.57%) stated they were likely, very likely or extremely likely to use the TAHK Framework in their future practice.

Consolidation of the TAHK Framework

The consultation findings highlighted that while the TAHK Framework was perceived to be useful, clinicians also wanted tools that provided specific guidance around its application in practice. This had previously been indicated by the greater uptake of process models in the existing allied health literature, than models which focused on determinants or implementation[5] As noted by Kielhofner, conceptual practice models are frameworks which are unique to an area of practice (in this case, allied health), and provide rationales, technologies and guidance for practice.[6] Conceptual practice models are familiar to allied health clinicians in their day to day work, and are a support which enables them to apply concepts practically. The development of rationales, technologies and guidance for practice therefore seemed to be the appropriate goal for the next phase of development.

However, the authors also wanted to support the measurement of KT outcomes, to discern which strategies and approaches were most effective. As noted by Chaudoir, Dugan and Barr,[7] KT is constrained by a lack of consensus around influential constructs and identifiable measures. While there was preliminary evidence that the TAHK Framework included constructs that were meaningful to allied health clinicians, the framework itself was not designed to measure outcomes. The authors decided to investigate the use of a benchmarking approach as a potential means of both guiding and measuring KT performance. The purpose of this phase of the framework's development was to enable outcome measurement and collect examples of how the TAHK aligned with allied health practice, and these findings are reported in full elsewhere.

The TAHK Benchmarking Tool was developed from an updated literature review about allied health KT, and the data collected in the first round of consultation. The tool itself comprised a printed document with eight sections. The first page included an introduction to the tool and some general information about the aims and process of benchmarking. Next, clinicians are asked to give their knowledge translation activity a title, identify the knowledge being translated and provide a 1-2 paragraph description of the activity. This qualitative information is intended to provide an overview of the knowledge translation activities being implemented across allied health.

The following four pages focused on the TAHK Framework domains. The factors under each domain were intended to reflect the important determinants of knowledge translation in relation to each domain, as identified in the literature and initial consultation with clinicians. Examples were provided for each factor, presented on a scale in order of increasing complexity, from the least complex (1) to the most complex (4). These factors and their associated examples (drawn from the literature review and prior data collection) are shown below, and clinicians were asked to tick or

shade the box that most closely corresponded to their approach. They were then encouraged to use a comment box at the end of the scale to describe the specific arrangements of their project or activity.

Given the diversity of disciplines and settings inherent in allied health, it would be impossible to construct a scale that accounted for all potential configurations of knowledge translation. At the bottom of each page of the TAHK Benchmarking Tool, three boxes were provided for further comments around what worked well (or was predicted to work well), what didn't work well (or was predicted to be problematic) and what they would like to do differently in their activity.

Table 2. Domains and Factors of the TAHK Benchmarking Tool

**Domain**	**Factor**	**1**	**2**	**3**	**4**
Doing	Timeframe	Take less than 3 months	Take 3 – 6 months	Take 6-12 months	Take more than 12 months
	Sources of Support	Use informal supports (i.e. no identified leader, ad hoc) within your department	Use general supports from outside your department (QA department, library)	Have the support of an identified project leader within your department	Have the support of an identified project leader within the broader organisation
	Systems and Procedures	Work within systems and procedures within your department	Work within 1 system and procedure within the broader organisation	Work within 2-3 system and procedure within the broader organisation	Work within 4+ system and procedure within the broader organisation
	Types of Knowledge	Use quantitative peer reviewed evidence only	Use quantitative and qualitative peer reviewed evidence only	Use all forms of peer reviewed evidence (including literature reviews, critiques and theoretical discussions)	Use all forms of evidence (including policies, patient feedback, learning from other services, etc)
Being	Knowledge Broker Involvement	Not work with a knowledge broker (skip next criterion)	Work with a knowledge broker specifically for this project/activity	Work with a knowledge broker on multiple projects/activities for a fixed term	Work with a knowledge broker permanently employed in the organisation
	Knowledge Broker Experience	Work with a knowledge broker who is not a health professional	Work with a knowledge broker from a different health profession	Work with a knowledge broker from your health profession	Work with a knowledge broker from your health profession and area of practice
	Knowledge Broker Qualifications	Work with a knowledge broker with no formal qualification (i.e. no health professional /	Work with a knowledge broker with health professional /	Work with a knowledge broker with health professional / discipline qualification and some	Work with a knowledge broker with health professional / discipline qualification and postgraduate

		disciplinary qualification)	discipline qualification or some research / quality assurance training	research / quality assurance training	qualifications (i.e. Certificate, Diploma, Masters, PhD)
Becoming	Capacity Building	Have no capacity building focus	Focused capacity building on educational initiatives (i.e. seminars, in-services)	Enabled limited participation in project/activity by workforce	Enabled regular participation in project/activity by workforce
	Organisational Strategy	Have no link to the organisation's overall strategy	Have broadly similar goals and focus as the organisation's overall strategy	Align with an explicit statement about knowledge translation / evidence-based practice in the organisation's overall strategy	Align with explicit policies and procedures about knowledge translation / evidence-based practice embedded in the organisation's overall strategy
	Discipline Focus	Have a discipline or specialist area specific focus	Have a broadly discipline specific focus	Have some relevance to other areas of practice	Have relevance to all areas of practice
Belonging	Workforce Participation	Not include participation by any member of the workforce	Include participation by only senior clinicians	Include participation by some members of the workforce	Include participation by all members of the workforce
	Patient / Carer Participation	Include no consultation with or participation by patients and/or carers	Include a one- off episode of consultation with or participation by patients and/or carers	Include a regular episodes of consultation with or participation by patients and/or carers	Embed consultation with or participation by patients and/or carers in all stages of performance
	Identification of Goals / Objectives	Have goals / objectives identified by the knowledge broker only	Have goals / objectives identified by the knowledge broker after discussion with the workforce	Have goals / objectives identified by the knowledge broker after discussion with the workforce and other stakeholders	Use a participatory process of goals / objectives including workforce, other stakeholders and patients and/or carers

The penultimate page of the TAHK Benchmarking Tool included an abbreviated version of the Evidence Based Practice Implementation Scale,[8] to capture the performance of knowledge translation in practice. This scale was originally developed in conjunction with an evidence-based practice belief scale, and contained 18 items.[8] The authors

decided to only utilise the implementation scale to measure enacted change (rather than the intended change which may be indicated by positive beliefs). This decision was guided by previous evidence that positive attitudes about evidence-based practice and knowledge translation do not automatically lead to its implementation in practice.[9]

The original Evidence Based Practice Implementation Scale was intended to measure all phases of evidence-based practice, and instructs clinicians to rate their performance of these behaviours over the past 8 weeks on a Likert scale. Previous psychometric testing on this original scale indicated excellent internal consistency, confirmation of the measurement of uni-dimensional constructs, and good criterion validity.[8] However, to meet the aims and focus of the TAHK Benchmarking Tool, eight items were discarded due to their explicit focus on research tasks or resources, given that the TAHK Framework encompasses the translation of more than just scientific research.

The final page contained an action plan, where clinicians identified the overall strengths of their activity, areas for further development, their goals for this on-going development and the resources required. They also identify a specific date for review of the action plan, to encourage iterative use of the tool.

Further Consultation with Knowledge Partners

A further professional development seminar was designed to provide an opportunity for allied health clinicians to trial a benchmarking tool based on the TAHK Framework. Modifications to the original ethics approvals were approved, and additional ethics approval was obtained from the university where the first two authors are employed (59/2016) to enable additional feedback from allied health academics to be collected. Each workshop ran for approximately 90 minutes, and they were attended by a total of 53 clinicians (including occupational therapists, physiotherapists, social workers, neuropsychologists and dieticians). Three occupational therapy academics also offered feedback on the tool.

Twelve clinicians and academics completed a measurement property survey for the benchmarking tool, which is considered an adequate sample for the measurement of face validity.[10] Seventy five percent of the sample agreed or strongly agreed that the benchmarking tool was a relevant and valid measure of influential factors for allied health KT, with the remaining participants expressing a neutral opinion. The overall mean content validity ration for the benchmarking tool was 0.73 ($P = .02$). Ayre and Scally[11] state that consensus from 10 participants is required for this sample size, and 11 of the 13 items on the benchmarking tool achieved this level. The two that did not both related to 'Being', and were once again related to knowledge broker attributes. These finding provided additional support for the validity of the TAHK Framework (given its reproduction within the tool), and also re-affirmed the factors related to the knowledge broker attributes were contentious.

At the conclusion of the quantitative feedback survey, participants were also invited to participate in a semi-structured interview to further explore their experience of using the benchmarking tool. Eight clinicians and academics were interviewed, which were transcribed verbatim and subjected to thematic analysis. Three overarching themes were derived from this data: "The Complexity of KT," "Focusing on Process rather than Outcomes," and "Tell Me More."

Participants frequently commented on the complexity of KT, and reported the TAHK framework increased their awareness of the multiple factors involved; *"It really highlights how much you need to think about"* (Interview 4). Some participants used the benchmarking tool to plan new activities, while others used it as a reflective resource for already completed activities. The framework was reported to be relevant to and supportive of both approaches "*I think it can be useful to kind of –stop and reflect – maybe after a year to kind of, okay, well, where are we at and if we're – wanting to continue on in some shape or form*" (Interview 1).

"Focusing on Process rather than Outcomes" refers to the strong theme from the data around wanting to use the TAHK Framework as a planning and evaluation resource, rather than as a basis for measuring outcomes. Nor did participants want to use the TAHK Framework for benchmarking their KT activities against those undertaken by allied health colleagues; "*I need to understand my project first before I think about other ones"* (Interview 3). Their focus was solely on their own activities rather than learning from others, possibly because they could not envision how such information might be presented.

Finally, most of the participants stated that they wanted more information and support about KT to enable them to use the TAHK Framework more effectively. "*Training should focus on understanding exactly what is required …. Once this has been grasped, it is relatively easy to use*" (Interview 7). Despite definitions being provided for some key terms, participants wanted additional explanation, resources and tools such as worked case studies, structured group activities and handouts for future reference. From this iterative process, in close partnership with allied health clinicians, managers and academics, the TAHK Framework was finalised and will now be described in detail.

References

1. Hitch D, Pepin G, Stagnitti K. The Pan Occupational Paradigm: Development and Key Concepts. *Scandinavian Journal of Occupational Therapy.* 2017;in press.
2. Hitch D, Pepin G, Stagnitti K. In the footsteps of Wilcock, Part One: The evolution of doing, being, becoming and belonging. *Occupational Therapy in Health Care.* 2014;28(3):231-246.
3. Bucknall T, Hitch D. Connections, Communication and Collaboration in Healthcare's Complex Adaptive Systems. Comment on "Using Complexity and Network Concepts to Inform Healthcare Knowledge Translation". *INternational Journal of Health Policy and Management.* 2017;6:Early online.

4. Forhan M, Law M. An Evaluation of a Workshop about Obesity Designed for Occupational Therapists. *Canadian Journal of Occupational Therapy.* 2009;76(5):351-358.
5. Nilsen P. Making sense of implementation theories, models and frameworks. *Implementation Science.* 2015;10:53.
6. Kielhofner G. *Conceptual foundations of occupational therapy* 4th ed. Practice: F.A. Davis Co; 2009.
7. Chaudoir S, Dugan A, Barr C. Measuring factors affecting implementation of health innovations: a systematic review of structural, organizational, provider, patient, and innovation level measures. *Implementation Science.* 2013;8:22.
8. Melnyk BM, Fineout-Overholt E, Mays MZ. The evidence-based practice beliefs and implementation scales: psychometric properties of two new instruments. *Worldviews on Evidence Based Nursing.* 2008;5(4):208-216.
9. Hitch D. Attitudes of mental health occupational therapists toward evidence-based practice: Attitudes des ergothérapeutes travaillant en santé mentale face à la pratique fondée sur les faits scientifiques. *Canadian Journal of Occupational Therapy.* 2015;83(1):27-32. doi:10.1177/0008417415583108
10. Mokkink L, Terwee C, Patrick D, et al. The COSMIN Checklist Manual. In. Amsterdam: VU University Medical Center; 2012.
11. Ayre C, Scally A. Critical Values for Lawshe's Content Validity Ratio: Revisiting the Original Methods of Calculation. *Measurement and Evaluation in Counseling and Development.* 2014;47(1):79-86.
